# Effect of the dilution rate on microbial competition: r-strategist can win over k-strategist at low substrate concentration

**DOI:** 10.1371/journal.pone.0172785

**Published:** 2017-03-23

**Authors:** Mari.-K. H. Winkler, Pieter Boets, Birk Hahne, Peter Goethals, Eveline I. P. Volcke

**Affiliations:** 1 Department of Biosystems Engineering, Ghent University, Gent, Belgium; 2 Department of Civil and Environmental Engineering, University of Washington, Seattle, Washington, United States of America; 3 Department of Applied Ecology and Biotechnology, Ghent University, Gent, Belgium; University of Notre Dame, UNITED STATES

## Abstract

The conditions present in both *in vitro* and *in vivo* ecosystems determine the microbial population harbouring it. One commonly accepted theory is that a species with a high substrate affinity and low growth rate (k-strategist) will win the competition against a second species with a lower substrate affinity and higher growth rate (r-strategist) if both species are subjected to low substrate concentrations. In this study two nitrite oxidizing bacteria (NOB), *Nitrospira defluvii* (k-strategist) and *Nitrobacter vulgaris* (r-strategist), were cultivated in a continuous reactor systems. The minimal hydraulic retention time (HRT) required for maintaining the slower growing *Nitrospira* was first determined. A reactor containing *Nitrobacter* was set to the same HRT and *Nitrospira* was injected to evaluate the effect of the dilution rate on the competition between both species. By following the microbial population dynamics with qPCR analysis, it was shown that not only the substrate affinity drives the competition between k- and r-strategists but also the dilution rate. Experimental data and numerical simulations both revealed that the washout of *Nitrobacter* was significantly delayed at dilution rates close to the μmax of *Nitrospira*. The competition could be even reverted towards *Nitrobacter* (r-strategist) despite of low nitrite concentrations and dilution rates lower than the μmax of *Nitrospira*.

## Introduction

In microbiology the competition of two species for one substrate is driven by their substrate affinity and their doubling time [1, 2]. An example is given by two common genera of nitrite oxidizing bacteria (NOB), namely *Nitrospira* (k-strategist) and *Nitrobacter* (r-strategist). *Nitrospira* possess a low maximum specific growth rate, but is well-adapted to low nitrite concentrations, whereas *Nitrobacter* is known to be a relatively fast-growing NOB with low affinities to nitrite [3, 4]. Since *Nitrospira spp*. and *Nitrobacter spp*. both use nitrite as substrate for growth, only the most adapted species will win the competition for nitrite and will hence outcompete the other one [5, 6]. NOB are vital organisms in the global nitrogen cycle [7] and play a key role in wastewater treatment systems. In the past it was thought that the predominant species responsible for nitrite oxidation in waste water treatment plants (wwtps) was *Nitrobacter spp*., since it was found in wwtp all over the world [8]. Nonetheless, molecular analysis, completed in various plants, showed that only a relatively small amount of *Nitrobacter spp*. were present in wwtps, while the dominant bacterium was *Nitrospira spp*. [3, 9, 10]. Since in a wwtp nitrite concentrations are low it is reasonable that *Nitrospira* and not *Nitrobacter* is the major NOB. However, in natural environments such as in soils or biofilms k and r strategists are found both at the same time despite of low bulk substrate concentrations [11]. Their occurrence can be explained by different (high or low) substrate profiles within a biofilm, which impact the competition between k- and r-strategists [12]. While there are several studies considering the effect of substrate concentration on species-species competition [1–6], the effect of the retention time has earned much less attention. Not only in engineered systems the hydraulic retention time is of high importance, but also in freshwater bodies and in the oceans where the HRT can be two days up to many hundred years [13, 14]. A study has shown that in a reactor running at low substrate availability and containing *Nitrobacter* and *Nitrospira*, the washout of *Nitrobacter* was delayed [15]. In their experiment, the effect of the HRT was not further investigated but it was assumed *a priori* that the winner would always be the species with the highest affinity for the limiting substrate. Nevertheless, in 1980, Hansen and Hubell established a theory, in which the competition of two species was described at a known limiting substrate concentration and at a certain dilution rate. Their study suggested that the dilution rate can revert the competition towards the r-strategist even at low substrate concentrations [16].

In this study, the effect of the dilution rate (retention time) on the competition of *Nitrobacter* and *Nitrospira* was evaluated at low substrate concentrations by means of dedicated experiments and numerical simulations. The aim was to show that not only the substrate concentration and hence the nitrite affinity constant will drive the competition between two species, but also the dilution rate, leading to a delayed washout of *Nitrobacter* and in some cases even to the unexpected dominance of *Nitrobacter* at low nitrite concentrations.

## Material and methods

### Cultivation of cultures

The strains used in this experiment were *Nitrobacter vulgaris* AB1 and *Nitrospira defluvii* A17 and the strains were provided from Hamburg University, Germany. Both bacteria were cultivated in batch flasks in a mineral salt freshwater medium for oligotrophic growth [17] and were used to inoculate the reactors (2.3L). The autoclaved reactors were fed with 230 mgNO_2_-N l^-1^ day^-1^, the pH was kept at pH 7, the temperature was controlled at 28°C a temperature controlled water jacket and DO at 7 mgO_2_ l^-1^. Baffles were used to assure good liquid gas transfer. Contamination was tested on a regular basis using Tryptone Soy agar CM0131 (Thermo Scientific). Nitrate and nitrite were measured with a spectrophotometer.

### Washout of Nitrospira defluvii

In order to determine the effect of the washout rate on interspecies competition, the minimal retention time needed to keep *Nitrospira defluvii* in the system was evaluated in the highly enriched *Nitrospira defluvii* reactor. The dilution rate was increased in a step-wise manner from 0.55 to 0.7 day^-1^ and the nitrite concentration was determined on a daily basis to follow nitrite accumulation over time. An increase of nitrite concentrations to values equal to the influent nitrite concentrations was evaluated as washout; the corresponding dilution rate was identified as the growth rate of *Nitrospira defluvii*.

### Competition experiment

The dilution rate of the *Nitrobacter* reactor was set to 0.58 days^-1^, corresponding with a value just below the minimal HRT needed to avoid the washout of *Nitrospira* as determined in a separate test (S5 Table). Both reactors were run with the same pump (1 for influent and 1 for the effluent), equipped with two identical pump heads, to ensure exactly the same dilution rate. The injection of *Nitrospira defluvii* into the *Nitrobacter vulgaris* reactor was conducted by spiking the culture through a sterile septum with a sterile needle into the reactor. The cells were counted and the amount of the injected culture was based on bacterial numbers rather than on the liquid volume. Cell counting was performed using a Bürker counting chamber (Marienfeld—Superior) and a light microscope (BX41—Olympus).

### DNA extraction and polymerase chain reaction (qPCR)

Samples were obtained from the effluent (600ml) and spun down at 4000 rpm for 20 minutes (Thermo Scientific Megafuge), the supernatant was discarded and the gained cell pellet was stored at -80°C until further usage. DNA extraction was based on the method of Niemann [18]. The samples were re-suspended in 20 μL alkaline lysis buffer, consisting of deionized water, 2.5% SDS solution (10%), and 5% sodium hydroxide solution (1mol l^-1^) and were kept in a warm water bath at 95°C for 15 minutes, followed by a cooling step on crushed ice for 10 minutes. The samples were centrifuged for 30 seconds at 13000 rpm and the pellet re-suspended in 180 μL deionized water.

In addition to the alkaline lysis, a phenol extraction and ethanol precipitation was completed to remove possible contaminants from the extracted DNA [19]. A phenol:chloroform:isoamyl solution (25:24:1), provided by Sigma-Aldrich, was added in an equal sample volume and vortexed vigorously for ca. 10 seconds. Subsequently, the samples were centrifuged at 13000 rpm for 2 minutes to separate the phases. The upper aqueous phase was transferred into a new tube while the lower organic phase was discarded. This step was repeated 3 times. In order to precipitate the extracted DNA an ethanol precipitation was accomplished. One tenth of the sample volume of 3 M sodium acetate solution (pH 5.4) was added. Then 2 volumes of ice cold 100% ethanol were added and well mixed. The mixture was stored at -80°C overnight and then spun down in a precooled microcentrifuge at 4°C at 14000 rpm for 30 minutes. The supernatant was removed and the pellet was air dried to remove excess ethanol and finally re-suspended in 200 μL DNA free water. Primers and PCR conditions are listed in S1 Table. Primers were checked in the ARB database as well as in the Ribosomal Database Project (RDP). All samples were measured in triplicates. The extracted DNA was used for a qPCR procedure with a variable primer concentration (S1 Table.) and 25 μM iQ SYBR^®^ Green Supermix (BIO-RAD Laboratories, USA). A “StepOne—Real Time PCR System” by Applied Biosystems was used to run the qPCR assays. All primers were optimized with a gradient qPCR. The resulting conditions, primer concentrations are listed in S1 Table. To determine the concentration of the extracted DNA the adsorption was measured with a NanoDrop2000-UV-VIS-Spektrophotometer (Thermo Scientific) at a wavelength of 260 nm. A 260/280 coefficient of 1.8 and a 260/230 coefficient of 2.0–2.2 were considered as pure DNA. Values below these ratios indicated pollution and were not considered for further analysis [20]. qPCR was performed on the pure cultures (C_T(*ref)*_*)* before the inoculation of the second culture (C_T(*target)*_) in order to have a zero value and to check if the reactors were contaminated. In order to compare the ΔC_T_ values between samples the following equation Δ*C*_*T*_ = *C*_*T*(*ref*)_ − *C*_*T*(*target*)_ was used [21]. For the calculations of the bacterial ratios, the approach from Winkler et al. (2011) was used. The highest ratio (pure culture) was designated as 1 and the ratios for the subsequent experiments were calculated as a fraction to achieve values between 1 (pure *Nitrobacter* culture) and 0 (no *Nitrobacter* present).

### Critical parameter J

In 1980 Hansen and Hubell experimentally proved the theory for resource based competition by showing that if multiple microbial strains compete in a continuous culture for the same limiting substrate, only one strain survives [16]. The theory is based on the quantity $\;{\text{J}}_{\text{i}}={\text{k}}_{\text{si}}\left(\frac{\text{D}}{\text{ri}}\right)$ and claims that if several strains compete for a single substrate, that the strain (i) with the lowest half saturation coefficient (k_Si_) on the limiting substrate (S), at the given intrinsic rate of increase (r_i_), as quantified by the smallest value for J, k will win the competition against the other species. The intrinsic rate of increase (r_i_) of strain i is defined as the difference between the growth rate (μ_i_) of the strain and the given dilution rate (D) [16]: *r*_*i*_ = (*μ*_*i*_ − D) with $\mu_{i}=\mu_{max}\frac{S_{O2}}{K_{O_{2}+}S_{O_{2}}}$ [22]. Dilution rates higher than the growth rate of the bacterium, correspond in the negative J value and lead to washout. In this study, the J criterion was applied to evaluate and compare the effect of the dilution rate on the competition between *Nitrobacter* and *Nitrospira*. The critical parameter J corresponding with the experimental conditions, was calculated and a sensitivity analysis was conducted to assess the effect of the dilution rate. For the nitrite affinity constant values of 1.5 and 0.21 mgNO_2_-N l^-1^ as well as maximum specific growth rates of 1.6 and 1 day^-1^ were used for *Nitrobacter*, whereas the values for *Nitrospira* were kept constant (0.12 mgNO_2_-N l^-1^ and 0.67 day^-1^), respectively (Table 1).

**Table 1:** Literature values reported for maximum specific growth rates and nitrite affinity constants for *Nitrobacter spp*. and *Nitrospira spp*.

***Nitrobacter***				
**Maximum growth rate**	reference value	conver. to 301K ^(1)^	unit	Reference
***Nitrobacter***	0.48	0.65	d^-1^	[23]
***Nitrobacter winogradskyi***	1.60	1.60	d^-1^	[24]
***Nitrobacter agelis***	0.86	1.17	d^-1^	[25]
***Nitrobacter spp***	0.67	0.9	d^-1^	[26]
**Average**	0.90	1		
**Nitrite affinity constant**				
***Nitrobacter spp***	1.50		mgNO2-N L^-1^	[23]
***Nitrobacter agelis***	2.80		mgNO2-N L^-1^	[27]
***Nitrobacter spp***	0.21		mgNO2-N L^-1^	[28]
**Average**	1.5		mgNO2-N L^-1^	
***Nitrospira***				
**Maximum growth rate**	reference value	conver. to 301K ^(1)^	unit	Reference
***Nitrospira spp***	0.50	0.68	d^-1^	[29]
***Nitrospira moscoviensis***	0.50	0.68	d^-1^	[30]
***Nitrospira devluvii***	this study	0.67	d^-1^	This study
**Average**	0.59	0.67	d^-1^	
**Nitrite affinity constant**				
***Nitrospira spp***	0.12		mgNO2-N L^-1^	[31]
***Nitrospira spp***	0.22		mgNO2-N L^-1^	[31]
***Nitrospira spp***	0.15		mgNO2-N L^-1^	[31]
***Nitrospira spp***	0.14		mgNO2-N L^-1^	[6]
	0.16		mgNO2-N L^-1^	

^(1)^ Conversion given in Hellinga et al 1999 through the relationship $\;\mu_{max}^{NOB}\left(T\right)=\mu_{max}^{NOB}\left(T_{ref}\right)\cdot \text{exp}\left(\frac{E_{a}^{NOB}\cdot (T-T_{ref)}}{R\cdot T\cdot T_{ref}}\right)$

### Model set-up development

A model including the competition between *Nitrobacter* and *Nitrospira* was implemented in the Aquasim software [32]. According to the r- and k-selection theory [1], *Nitrospira* (Nsp) is represented as k-strategist, with a low growth rate but a high affinity for nitrite, whereas *Nitrobacter* (Nb) is represented as r-strategist, with a high growth rate and low affinity for nitrite. The affinity constants and growth rates were set according to reported literature values (Table 1). The overall model stoichiometry and kinetics are summarized in the Appendix (S2 and S3 Tables, respectively), as are the corresponding parameter values (S4 Table). The oxygen was set to the same constant value as in the experiment. Since the HRT was close to the maximum specific growth rates of both bacteria, decay was neglected. Monod kinetics were used to describe the dependency of growth on nitrite and oxygen of both NOB [33]. Ammonium was used as nitrogen source for biomass growth. For the simulations, the same average literature values (given in Table 1) as for the calculations of the critical parameter J were used.

## Results

### Minimal hydraulic retention time for Nitrospira defluvii

The purpose of this study was to evaluate the effect of the dilution rate on the competition between a k-strategist (*Nitrospira*) and an r-strategist (*Nitrobacter)*. Since the knowledge of maximum growth rate of *Nitrospira defluvii* was highly important for this experiment, its value was estimated by determining the minimal hydraulic retention time before washout occurred, by gradually increasing the flow rate (S5 Table). The minimum dilution rate of *Nitrospira* before washout was experimentally determined to lie between 0.6–0.67 day^-1^, which is in line with literature values [29, 30] (Table 1 and S5 Table). Therefore, all reactors were run with a dilution rate of 0.6 day^-1^.

### Experimental validation of the dynamic competition behaviour—Influence of dilution rate

Two separate reactors were run and as expected the number of cells of *Nitrobacter* was lower (1.5*10^8^ cells mL^-1^) than that of *Nitrospira* (2*10^9^ cells mL^-1^) due to a higher yield coefficient of the latter one [26, 31]. The reactor containing *Nitrobacter* was inoculated with *Nitrospira* based on cell concentration with a ratio 0.75:0.25 and this ratio was also used as starting condition in the model. In order to compare the simulation results with molecular data from the qPCR assay, both results were expressed in a ratio of Nb: (Nsp+Nb). A ratio of 1 meant that a pure culture of *Nitrobacter* was present and a ratio of 0 that *Nitrospira* dominated the system. Accordingly, at a ratio of 0.5 both bacteria were present in equal amounts.

**Fig 1:**
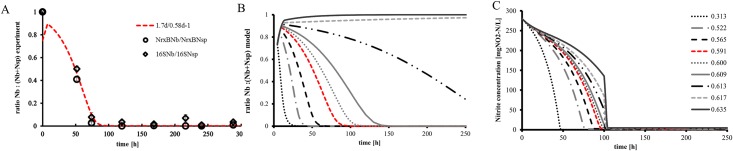
A) Comparison between measured ratios (qpcr results) of *Nitrobacter*/*Nitrospira* in terms of their functional genes (NrxbNb/ NrxBNsp) (◌) and 16SDNA (◊) and corresponding simulation result at a hydraulic retention time of 1.7 days (corresponding to μmax of 0.58d^-1^; red dashed line). The experiment and the simulation were based on an inoculation ratio of (0.75:0.25). B) Influence of the dilution rate on the ratios of *Nitrobacter*/*Nitrospira* Nb: (Nsp+Nb). C) Corresponding substrate concentration to biomass profile at given dilution rate. All graphs were determined for constant parameter values for *Nitrospira* (μ_max_ = 0.67d^-1^, K_s_ = 0.21 mgNO_2_-N l^-1^) and *Nitrobacter* (μ_max_ = 1d^-1^, K_s_ = 1.5mgNO_2_-N l^-1^).

The ratios calculated for 16S and functional primers (NxrB) delivered comparable results since both measuring points overlap (Fig 1A). The simulations started with the Nb: (Nsp+Nb) ratio of 0.75:0.25 as it was chosen in the experimental design as mentioned in this section above (Fig 1A). The dilution rate (~0.6 day^-1^) applied in the experiment perfectly corresponded with the simulations conducted at a similar dilution rate (0.58 day^-1^, Fig 1A). The minor difference between the dilution rate on the experiment and the simulations (0.02 day^-1^) could be explained by inaccuracies in the pump frequency. The validated model was subsequently applied to study the effect of the dilution rate on the competition at given nitrite affinity constants and growth rates (Fig 1B). At low dilution rates (HRT of >3 days) *Nitrospira* out-competed *Nitrobacter* instantaneously. However, when the dilution rate was set close to the maximal growth rate of *Nitrospira* (D≈0.67 day^-1^) small changes had a big impact on the time needed for complete washout of *Nitrobacter*. The time needed for washout of *Nitrobacter* increased from 100 hours to over 1000 hours for dilution rates from 0.58 to 0.63 d^-1^ (Fig 1B). For hydraulic retention times higher than 0.64 day^-1^ *Nitrospira* was washed out of the reactor, although a pure culture of *Nitrospira* could have remained in the system until the dilution rate became higher than the maximal possible growth rate of 0.67 day^-1^ (Table 1 and S5 Table). Pure culture studies on a single substrate, such as conducted here, clearly demonstrate the role of the dilution rate in a continuously fed reactor on the competition between *Nitrospira* and *Nitrobacter*.

### The effect of the dilution rate on microbial competition

**Fig 2:**
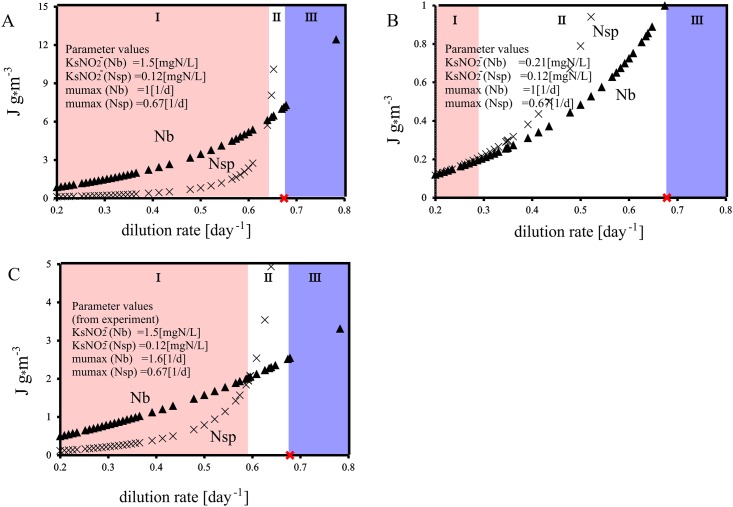
Simulation result of the critical parameter J between *Nitrospira* (x) and *Nitrobacter* (▲) in terms of the dilution rate. Red (**x**) indicates the dilution rate above which washout of the pure *Nitrospira* culture occurred. The bacterium with the lower J will win the competition. The value of the maximum specific growth rate and nitrite affinity constant of both bacteria are given in each graph (A,B,C). Results in panel A are based on values from the experiment. In panel B) all values were kept the same as in panel A except the Ks value of *Nitrobacter*, which was set smaller (higher affinity). In panel C all values were kept the same as in panel A but the growth rate of *Nitrobacter*, which was defined to be larger. In zone I *Nitrospira* wins the competition. Zone II indicates the range of dilution rates at which *Nitrobacter* (Nb) wins the competition despite a lower affinity for nitrite, and given that the prevailing dilution rate was lower than the μmax of *Nitrospira* (Nsp). From the moment at which the dilution rate exceeds the μmax of *Nitrospira* (bold x), *Nitrospira* is washout out and *Nitrobacter* dominates the system (zone III).

Simulations on the concentration in the reactor showed that the delayed washout of *Nitrobacter* was coupled to the nitrite concentrations in the reactor (Fig 1C). However, the measurements did not show nitrite in the effluent, which is possibly due to some biomass growth on the reactor walls, hence decreasing nitrite to low levels. In order to inspect the competition at limiting concentrations the critical parameter J was used. The parameter J determines the competition outcome and is governed by the species substrate affinity and maximum growth rate as well as by the prevailing dilution rate. The higher the growth rate and the lower the substrate affinity, the lower the critical parameter J (at a given dilution rate) will be. The species with the lowest J will win the competition [16]. *Nitrobacter* can only win at low substrate concentrations if its J value is lower than the one of *Nitrospira*. The J values for both species are increasing towards higher dilution rates, and are the highest close to the point of the dilution rate corresponding to their specific growth rate (Fig 2). At the point where the dilution rate equals the maximum growth rate, J becomes negative (not displayed) indicating washout. Fig 2A displays the effect of the dilution rate for typical growth rates and affinity constants for *Nitrospira* and *Nitrobacter* (Table 1). The range of dilution rates at which *Nitrobacter* outcompeted *Nitrospira* despite of low substrate conditions and although the retention time was high enough to retain *Nitrospira* as a single culture in the system, was very narrow (zone II).

A sensitivity analysis was conducted to assess the effect of nitrite affinity (Fig 2B) and the maximum growth rate (Fig 2C) on the competition outcome in terms of the different dilution rates. The effect of changing the growth rate to the highest reported literature value of *Nitrobacter* (1.6 day^-1^, Table 1) did show an effect, but only in a narrow range (Fig 1C, zone II). *Nitrobacter* won the competition at a dilution rate corresponding to a μmax of 0.6 day^-1^ even though the washout for a single species experiment (with *Nitrospira* only) would have led to a washout only when the dilution rate exceeded the maximal growth rate (0.67 day^-1^, Fig 2). However, the range in which the dilution rate could reverse and favour the competition towards *Nitrobacter*, was similar to the experimental conditions (Fig 2A, zone II), very narrow (0.6–0.67 day^-1^) and hence very close to the μmax of *Nitrospira* (0.67 day^-1^). The value of the affinity constant had a more severe effect on the competition (Fig 2B). When applying the lowest nitrite affinity constant reported in literature for *Nitrobacter* (Table 1) the washout of *Nitrospira* occurred at significantly lower dilution rates (0.3 day^-1^) than the μmax of *Nitrospira* (0.67 day^-1^) (Fig 2B, zone II). The J values for both organisms were very close to one another at all dilution rates, indicating a strong competition.

## Discussion

The results presented here demonstrate that the dilution rate is of crucial importance regarding the competition between k and r strategists (Figs 1 and 2). The experimentally determined washout of *Nitrobacter* at higher dilution rates matched the simulation results very well, hence providing a reliable starting point for further simulation studies evaluating for various operational strategies, which would have been very time consuming to be tested experimentally. In this study, the chosen dilution rate was not close enough to the μmax of *Nitrospira* in order to provoke its washout by competition, which is nevertheless possible as shown in Figs 1 and 2. A washout of *Nitrospira* at a dilution rate close to its μmax would have been easily interpreted as washout due to high dilution rate (e.g. due to inaccuracies of the pumps). Our simulation results show that the sludge retention time (SRT) has a big impact on the substrate concentrations, hence severely influencing the competition between *Nitrospira* and *Nitrobacter*. Under these circumstances it was shown here that the bacterium with the highest substrate affinity (*Nitrospira*) does not necessarily win the competition at low substrate concentrations as commonly perceived in literature [1–6]. The dilution rate is crucial especially when the difference between the value of the affinity constants of two species competing for the same substrate is small (Fig 1B). Under these circumstances will the competition take more time to reach steady state (washout of *Nitrobacter*) and can even be reverted from one to another species as shown in the simulation studies (Figs 1 and 2B). In an earlier study based on the competition of *Nitrobacter* and *Nitrospira* at low substrate concentrations, a fast washout of *Nitrobacter* did not occur [15], which might have been a delayed washout of *Nitrobacter* over *Nitrospira* as shown in our study (Fig 2B).

In a wastewater treatment plant operated with flocs, the retention time of bacteria (sludge retention time, SRT) can be controlled independently from the hydraulic retention time (HRT, being the inverse of the dilution rate). The J criterion can also be applied in systems in which the SRT is not equal to the HRT but still the same for all bacteria [22, 34]. Nature however, is often more complex, for instance individual species in biofilms are characterized by different SRTs. The straightforward criteria to determine the competition outcome, such as based on the J-value, can therefore not easily be formulated in biofilm systems [35]. Besides, biofilms display concentration gradients in terms of biofilm depth in which different substrate concentrations select for r and k strategists at the same time [12].

Earlier modelling studies, investigating the growth of bacteria on multiple substrates, have shown that the coexistence of several species at steady state is possible due to niche differentiation by substrate inhibition, even when all the organisms strongly prefer one substrate [36]. In addition, it is known that *Nitrobacter* is capable of mixotrophic growth, hence using other substrates than nitrite [37] increasing the complexity of a clear determination of the winning strategy of k and r-strategists [38].

Despite of the complexity of substrate competition between different species, the outcome of this can study give an explanation for situations in which a r-strategist is dominating a system at low substrate concentrations. In this study, it was shown that the dilution rate within an ecosystem creates a selection pressure favouring the most adapted species under given environmental conditions, hence out-competing other less adapted species [39, 40], which is in this case unexpectedly r-strategists (*Nitrobacter*) winning the competition at low substrate concentrations. In biotechnological processes the bacterial retention time is often uncoupled from dilution rate by using biofilm systems, which retain high biomass concentrations. However, in these biofilm systems suspended biomass is present as well and the selection for different nitrifying populations based on biofilms and/or suspended biomass and the impact on ecophysiological population dynamics is poorly understood. Therefore one must better comprehend what conditions bacteria experience when growing in suspension versus in a biofilm. Recent studies insinuate that suspended biomass plays different functional roles in biofilm systems, and that suspended biomass and biofilms can coexists in a single system [41–44]. Since biofilm reactor systems can remove substrate concentrations to low levels at high dilution rates the selection pressure of the dilution rate will severely drive population dynamics in the suspended fraction [45, 46], and might lead to the dominance of r-strategists at low substrate concentrations. In a system that runs at high dilution rates a good knowledge of the μmax and affinity constants are of prime importance for accurate simulation and successful reactor operation. One must take care not only to fit a model with experimental data by changing affinity constants and growth rates only, but should also consider the correct hydraulic or sludge retention time. It is clear that a model can describe a system only as exact as the input data are [47] and a validation is hence vital (Fig 2A).

In conclusion, it was demonstrated that not only the substrate concentrations but also the dilution rate in natural habitats and engineered systems drives interspecies competition and must be taken into consideration, while investigating the competition between k and r strategists.

## Supporting information

S1 Table Primers, qPCR conditions and primer concentrations used in this study. (DOCX)

S2 Table Stoichiometric matrix (*A*_*ij*_) of the model describing the growth of *Nitrobacter* (*X*_*Nb*_) and *Nitrospira* (*X*_*Nsp*_). The conversion rate (R_i_) of a component i is related to the process rates (ρ_j_) through *R*_*i*_ = ∑_*j*_ *A*_*ij*_ *ρ*_*j*_.
(DOCX)

S3 Table Reaction kinetics for *Nitrobacter* (Nb) and *Nitrospira* (Nsp). (DOCX)

S4 Table Stoichiometric and kinetic parameter values. (DOCX)

S5 Table Determination of the minimal necessary Hydraulic Retention Time (HRT) to avoid washout in two separate batch reactors containing *Nitrobacter* and *Nitrospira*. (DOCX)

## References

[1] Andrews JH, Harris RF. r- and K-Selection and Microbial Ecology In: Marshall KC, editor. Advances in Microbial Ecology. Advances in Microbial Ecology. 9: Springer US; 1986 p. 99–147.

[2] Dorodnikov M, Blagodatskaya E, Blagodatsky S, Fangmeier A, Kuzyakov Y. Stimulation of r- vs. K-selected microorganisms by elevated atmospheric CO2 depends on soil aggregate size. FEMS Microbiology Ecology. 2009;69(1):43–52. 10.1111/j.1574-6941.2009.00697.x 19453741

[3] Daims H, Nielsen JL, Nielsen PH, Schleifer K-H, Wagner M. In Situ Characterization of Nitrospira-Like Nitrite-Oxidizing Bacteria Active in Wastewater Treatment Plants. Applied Environmentral Microbiology. 2001;67(11):5273. 10.1128/AEM.67.11.5273-5284.2001 PMC93301 11679356

[4] Spieck E, Hartwig C, McCormack I, Maixner F, Wagner M, Lipski A, et al Selective enrichment and molecular characterization of a previously uncultured Nitrospira-like bacterium from activated sludge. Environmental Microbiology. 2006;8(3):405–15. 10.1111/j.1462-2920.2005.00905.x 16478447

[5] Kim D-J, Kim S-H. Effect of nitrite concentration on the distribution and competition of nitrite-oxidizing bacteria in nitratation reactor systems and their kinetic characteristics. Water Research. 2006;40(5):887–94. 10.1016/j.watres.2005.12.023. 16460781

[6] Schramm A, de Beer D, van den Heuvel J, Ottengraf S, Amann R. Microscale distribution of populations and activities of Nitrosospira and Nitrospira spp. along a macroscale gradient in a nitrifying bioreactor: quantification by in situ hybridization and the use of microsensors. Applied and Environmental Microbiology. 1999;65(3690–3696). 10427067 10.1128/aem.65.8.3690-3696.1999 PMC91552

[7] Zehr JP, Kudela RM. Nitrogen Cycle of the Open Ocean: From Genes to Ecosystems In: Carlson CA, Giovannoni SJ, editors. Annual Review of Marine Science, Vol 3 Annual Review of Marine Science. 3. Palo Alto: Annual Reviews; 2011 p. 197–225. 10.1146/annurev-marine-120709-142819 21329204

[8] Halling-Sørensen B, Jørgensen SE. The removal of nitrogen compounds from wastewater. Elsevier, Amsterdam, The Netherlands 1993.

[9] Burrell P, Keller J, Blackall LL. Characterisation of the bacterial consortium involved in nitrite oxidation in activated sludge. Water Science and Technology. 1999;39(6):45–52. 10.1016/S0273-1223(99)00121-3.

[10] Burrell PC, K J., Blackall LL. Microbiology of a Nitrite-Oxidizing Bioreactor. Applied Environmentral Microbiology. 1998;64(5):1878–83. 10.1128/aem.64.5.1878-1883.1998 PMC106245 9572966

[11] Poly F, Wertz S, Brothier E, Degrange V. First exploration of Nitrobacter diversity in soils by a PCR cloning-sequencing approach targeting functional gene nxrA. FEMS Microbiology Ecology. 2008;63(1):132–40. 10.1111/j.1574-6941.2007.00404.x 18031541

[12] Winkler MK, Kleerebezem R, de Bruin LM, Verheijen P, Abbas B, Habermacher J, et al Microbial diversity differences within aerobic granular sludge and activated sludge flocs. Applied Microbiolology Biotechnology. 2013;97(16):7447–58. 10.1007/s00253-012-4472-7 23064482

[13] Hilton ABH, McGillivary DL, Adams EE. Residence time of freshwater in Bostons inner Harbor J Waterway, Port, Coastal, Ocean Eng 1998;124:82–9.

[14] Stuiver M, Quay PD, Ostlund HG. Abyssal water C-14 distribution and the age of the world oceans. Science. 1983;219(4586):849–51. 10.1126/science.219.4586.849 17780221

[15] Nogueira R, Melo LF. Competition between Nitrospira spp. and Nitrobacter spp. in nitrite-oxidizing bioreactors. Biotechnology and Bioengineering. 2006;95(1):169–75. 10.1002/bit.21004 16703620

[16] Hansen, Hubbell SP. Single-nutrient microbial competition: qualitative agreement between experimental and theoretically forecast outcomes. Science. 1980;207(4438):1491–3. 6767274 10.1126/science.6767274

[17] Spieck E, Lipski A. Cultivation, growth physiology and chemotaxonomy of nitrite-oxidizing bacteria. Methods in Enzymology. 2011;486. 10.1016/B978-0-12-381294-0.00005-5 21185433

[18] Niemann S, Pühler A, Tichy HV, Simon R, Selbitschka W. Evaluation of the resolving power of three different DNA fingerprinting methods to discriminate among isolates of a natural Rhizobium meliloti population. Journal of Applied Microbiology. 1997;82(4). 10.1046/j.1365-2672.1997.00141.x 9134721

[19] Ausubel FM, Brent R, Kingston RE, Moore DD, Seidman JG, Smith JA, et al Current Protocols in Molecular Biology. 3rd, editor. New York: John Wiley & Sons, Inc.; 1994.

[20] Scientific TF. NanoDrop 2000/2000c Spectrophotometer V1.0 User Manual. Wilmington, DE 19810 U.S.A.: Thermo Fisher Scientific; 2009.

[21] Zhang HY, Bao SM, Shou WL, Luan HX, Zhang Y, Feng X, et al Expression of matrix metalloproteinase-1 mRNA in peripheral blood mononuclear cells of systemic lupus erythematosus patients and its relationship with atherosclerosis. Chinese Med J-Peking. 2009;122(21):2593–7. 19951575

[22] Volcke EIP, Sanchez O, Steyer JP, Dabert P, Bernet N. Microbial population dynamics in nitrifying biofilm reactors: experimental evidence described by a simple model including interspecies competition. Process Biochem. 2008;43:1398–406.

[23] Vadivelu VM, Yuan Z, Fux C, Keller J. Stoichiometric and kinetic characterisation of Nitrobacter in mixed culture by decoupling the growth and energy generation processes. Biotechnology and Bioengineering. 2006;94(6):1176–88. 10.1002/bit.20956 16673416

[24] Boon B, Laudelout H. Kinetics of Nitrite Oxidation by Nitrobacter winogradskyi. Biochemistry Journal 1962;85(440). 10.1042/bj0850440 PMC1243762 14013807

[25] Tramper J, Grootjen DRJ. Operating performance of Nitrobacter agilis immobilized in carrageenan. Enzymatic Microbial Technology. 1986;8:477–80.

[26] Blackburne R, Vadivelu VM, Yuan Z, Keller J. Determination of Growth Rate and Yield of Nitrifying Bacteria by Measuring Carbon Dioxide Uptake Rate. Water Environment Research. 2007;79(12):2437–45. 18044361 10.2175/106143007x212139

[27] O'Kelly JO, Becker GE, Nason A. Characterization of the particulate nitrite oxidase and its component activities from the hemoautotrophic Nitrobacter agilis. Biochemica et Biophysica Acta 1997;409(45):951. 10.1016/0005-2728(70)90107-6 4394298

[28] Bock E, Koops H. The genus Nitrobacter and related genera In: Balows A, Trüper HG, Dworkin M, Harder W, Schleifer K-H (eds). The Prokaryotes, 2nd edn Springer-Verlag: New York pp 2302–2309. 1992.

[29] Kindaichi T, Kawano Y, Ito T, Satoh H, Okabe S. Population dynamics and in situ kinetics of nitrifying bacteria in autotrophic nitrifying biofilms as determined by real-time quantitative PCR. Biotechnology and Bioengieering. 2006;94(6):1111–21. 10.1002/bit.20926 16596626

[30] Ehrich S, Behrens D, Lebedeva E, Ludwig W, Bock E. A new obligately chemolithoautotrophic, nitrite-oxidizing bacterium,Nitrospira moscoviensis sp. nov. and its phylogenetic relationship. Archives of Microbiology. 1995;164(1):16–23. 7646315 10.1007/BF02568729

[31] Blackburne R, Vadivelu VM, Yuan Z, Keller J. Kinetic characterisation of an enriched Nitrospira culture with comparison to Nitrobacter. Water Research. 2007;41(14):3033–42. 10.1016/j.watres.2007.01.043. 17553540

[32] Reichert P. Aquasim—a Tool for Simulation and Data-Analysis of Aquatic Systems. Water Sci Technol. 1994;30(2):21–30.

[33] Monod J. The Growth of Bacterial Cultures. Annual Review of Microbiology. 1949;3(1):371–94.

[34] Hsu SB, Hubbell S, Waltman P. A mathematical theory for single-nutrient competition in continuous cultures of micro-organisms. SIAM J Appl Math. 1977;32(2):366–83.

[35] Vannecke TPW, Bernet N, Steyer JP, Volcke EIP. Modelling ammonium-oxidizing population shifts in a biofilm reactor. Water Science and Technology. 2014;61(1):208–16. 10.2166/wst.2013.701 24434989

[36] Yoon G, Klinzing G., Blanch H.W. Competition for Mixed Substrates by Microbial Populations. Biotechnology and Bioengieering. 1977;19(1193–1210). 10.1002/bit.260190809 884234

[37] Steinmüller W, Bock E. Growth of Nitrobacter in the Presence of Organic Matter. Archives of Microbiology. 1976;108:299–304. 821450 10.1007/BF00454856

[38] Winkler MKH, Bassin JP, Kleerebezem R, Sorokin DY, van Loosdrecht MCM. Unravelling the reasons for disproportion in the ratio of AOB and NOB in aerobic granular sludge. Applied Microbiology and Biotechnology. 2012;94(6):1657–66. 10.1007/s00253-012-4126-9 22573276 PMC3359442

[39] Baas-Becking LGM. Geologie of Inleiding Tot de Milieukunde. The Hague, The Netherlands: W. P. Van Stokum 1934.

[40] Beijerinck MW. De infusies en de ontdekking der backteriën. In Jaarboek van de Koninklijke Akademie van Wetenschappen, Müller, Amsterdam. 1913.

[41] Innerebner G, Insam H, Franke-Whittle IH, Wett B. Identification of anammox bacteria in a full-scale deammonification plant making use of anaerobic ammonia oxidation. Systematic and Applied Microbiology. 2007;30(5):408–12. 10.1016/j.syapm.2007.02.001 17399934

[42] Vlaeminck SE, Terada A, Smets BF, De Clippeleir H, Schaubroeck T, Bolea S, et al Aggregate size and architecture determine microbial activity balance for one-stage partial nitritation and anammox. Applied and Environmental Microbiology. 2010;76(3):900–9. 10.1128/AEM.02337-09 19948857 PMC2813012

[43] Winkler M-KH, Bassin JP, Kleerebezem R, de Bruin LMM, van den Brand TPH, van Loosdrecht MCM. Selective sludge removal in a segregated aerobic granular biomass system as a strategy to control PAO–GAO competition at high temperatures. Water Research. 2011;45(11):3291–9. 10.1016/j.watres.2011.03.024. 21513967

[44] Winkler M-KH, Kleerebezem R, Kuenen JG, Yang J, van Loosdrecht MCM. Segregation of Biomass in Cyclic Anaerobic/Aerobic Granular Sludge Allows the Enrichment of Anaerobic Ammonium Oxidizing Bacteria at Low Temperatures. Environmental Science & Technology. 2011;45(17):7330–7. 21744798 10.1021/es201388t

[45] Arrojo B, Mosquera-Corral A, Campos JL, Méndez R. Effects of mechanical stress on Anammox granules in a sequencing batch reactor (SBR). Journal of Biotechnology. 2006;123(4):453–63. 10.1016/j.jbiotec.2005.12.023. 16473427

[46] Vlaeminck SE, Cloetens LFF, De Clippeleir H, Carballa M, Verstraete W. Granular biomass capable of partial nitritation and anammox (Water Science and Technology 58(5) 1113–1120). Water Science and Technology2009. p. 609–17. 10.2166/wst.2008.731 18824812

[47] Kavetski D, Franks SW, Kuczera G. Confronting input uncertainty in environmental modelling. Water Science and Application. 2003;6:49–68.

