# Supplementary material for: Effect of the dilution rate on microbial competition: r-strategist can win over k-strategist at low substrate concentration
Source: PLoS One. 2017 Mar 23;12(3):e0172785. doi: 10.1371/journal.pone.0172785 (PMC5363889; doi:10.1371/journal.pone.0172785)
Supplement: S1 Table — (DOCX) [file pone.0172785.s001.docx]

| **S1 Table**. Primers, qPCR conditions and primer concentrations used in this study. | | | | |
| --- | --- | --- | --- | --- |
| **Primer** | **con. [µM]** | **qPCR conditions** | **Sequence (5′-3′)** | **Reference** |
| NTS 232F/  NTS 1200R | 0.2 | 95/5m, (95/40s, 55-48/30s, 72/60s)*35, 72/10min, 12/∞ | GCTCATGTCCTATCAGCTTG/ AGGCATAAAGGCCATGCTG | [[1](#_ENREF_1)] |
| NxrB F19/  NxrB R1237 | 0.2 | 95/5m, (95/40s, 55/30s, 72/60s)*35, 72/10min, 12/inf. | TGGCAACTGGGACGGAAGATG/  GTAGATCGGCTCTTCGACCTG | [[2](#_ENREF_2)] |
| NxrB F70/  NxrB R1431 | 0.2 | 95/5m, (95/40s, 56/30s, 72/30s)*35, 72/10min, 12/∞ | AAGACCTAYTTCAACTGGTC/  CGCTCCATCGGYGGAACMAC | [[3](#_ENREF_3)] |
| FGPS872/  FGPS 1269 | 0.2 | 95/3m, (95/60s, 50/60s, 72/60s)*35, 72/3min, 12/∞ | CTAAAACTCAAAGGAATTGA/  TTTTTTGAGATTTGCTAG | [[4](#_ENREF_4)] |
| Bac341F | 0.35 | 95°/6’ [95°/0.30’ 55°/0.4’ 52°/0.4’80°/0.25’] x40 | ACGGGAGGCAGCAG | [[5](#_ENREF_5)] |
| Bac905R | 0.35 | 52°/5’10°/∞ | TCTGTCATTGTAGCATCTTT | [[6](#_ENREF_6)] |
| Primers used and their concentrations as well as the standard used. qPCR conditions show temperature and cycle length for denaturing, annealing, elongation and cooling steps. | | | | |

**References**

1. Lim J, Do H, Shin SG, Hwang S. Primer and probe sets for group-specific quantification of the genera Nitrosomonas and Nitrosospira using real-time PCR. Biotechnology and Bioengineering. 2008;99(6):1374-83. doi: 10.1002/bit.21715.

2. Pester M, Maixner F, Berry D, Rattei T, Koch H, Lücker S, et al. NxrB encoding the beta subunit of nitrite oxidoreductase as functional and phylogenetic marker for nitrite-oxidizing Nitrospira. Environmental Microbiology. 2014:16(0):3055-71. doi: 10.1111/1462-2920.12300.

3. Koch H. Ecophysiological investigation of nitrite-oxidizing bacteria of the genus Nitrospira. Wien: Universität Wien; 2009.

4. Degrange V, Bardin R. Detection and counting of Nitrobacter populations in soil by PCR. Applied and Environmental Microbiology. 1995;61(6):2093-8.

5. Muyzer G, Dewaal EC, Uitterlinden AG. Profiling of complex microbial-popultions by denaturing gradient Gel-electrophoresis ofpolymerase chain reaction-amplified genes coding for 16S ribosomal-RNA. Applied and Environmental Microbiology. 1993;59:695-700.

6. Weisburg WG, Barns SM, Pelletier DA, Lane DJ. 16S ribosomal DNA amplification for phylogenetic study. Journal of Bacteriology. 1991;173(2):697-703.
