# Supplementary material for: Effect of the dilution rate on microbial competition: r-strategist can win over k-strategist at low substrate concentration
Source: PLoS One. 2017 Mar 23;12(3):e0172785. doi: 10.1371/journal.pone.0172785 (PMC5363889; doi:10.1371/journal.pone.0172785)
Supplement: S2 Table — The conversion rate (Ri) of a component i is related to the process rates (ρj) through Ri = ∑j Aij ρj. (DOCX) [file pone.0172785.s002.docx]

**S2 Table.** Stoichiometric matrix ($A_{ij}$) of the model describing the growth of *Nitrobacter* ($X_{Nb}$) and *Nitrospira* ($X_{Nsp}$).The conversion rate (R_i_) of a component i is related to the process rates (ρ_j_) through $R_{i}=\sum_{j} A_{ij}\rho_{j}$.

| A_ij_ | i component →  j process **↓** | S_NH_  ammonium  [g N.m^‑3^] | S_NO2-_  nitrite  [g N.m^‑3^] | S_NO3_  nitrate  [g N.m^‑3^] | S_O2_  oxygen  [g O_2_.m^‑3^] | X_Nb_  *Nitrobacter*  [g COD.m^‑3^] | | X_Nsp_  *Nitrospira*  [g COD.m^‑3^] | | Process  rate ρ_j_ | |
| --- | --- | --- | --- | --- | --- | --- | --- | --- | --- | --- | --- |
| Growth Nitrobacter (Nb) | | -i_NXB_ | -1/Y_Nb_ | 1/Y_Nb_ | 1-1.14/Y_Nb_ | 1 |  | | ρ_G,Nb_ | |  |
| Growth Nitrospira (Nsp) | | -i_NXB_ | -1/Y_Nsp_ | 1/Y_Nsp_ | 1-1.14/Y_Nsp_ |  | 1 | | ρ_G,Nsp_ | |  |
| Composition matrix | |  |  |  |  |  |  | |  | |  |
| g COD/unit comp | |  | -3.43 | -4.57 | -1 | 1 | 1 | |  | |  |
| g N/unit comp | | 1 | 1 | 1 |  | i_NXB_ | i_NXB_ | |  | |  |
