# Supplementary material for: Effect of the dilution rate on microbial competition: r-strategist can win over k-strategist at low substrate concentration
Source: PLoS One. 2017 Mar 23;12(3):e0172785. doi: 10.1371/journal.pone.0172785 (PMC5363889; doi:10.1371/journal.pone.0172785)
Supplement: S3 Table — (DOCX) [file pone.0172785.s003.docx]

**S3** **Table**. Reaction kinetics for *Nitrobacter* (Nb) and *Nitrospira* (Nsp).

| j process **↓** |  |
| --- | --- |
| ρ_G,Nb_ |  |
| ρ_G,Nsp_ |  |
