# Supplementary material for: Effect of the dilution rate on microbial competition: r-strategist can win over k-strategist at low substrate concentration
Source: PLoS One. 2017 Mar 23;12(3):e0172785. doi: 10.1371/journal.pone.0172785 (PMC5363889; doi:10.1371/journal.pone.0172785)
Supplement: S4 Table — (DOCX) [file pone.0172785.s004.docx]

**S4 Table.** Stoichiometric and kinetic parameter values.

| Parameter | Description | Value | Unit | Reference |
| --- | --- | --- | --- | --- |
| **Stoichiometric parameters** | | | | |
| i_NXB_ | Nitrogen fraction in biomass | 0.17 | g N.(g COD)^-1^ | [[1](#_ENREF_1)] |
| Y_Nb_ | Yield coefficient of Nb | 0.057 | g COD.(g N)^-1^ | [[2](#_ENREF_2), [3](#_ENREF_3)]^(1)^ |
| Y_Nsp_ | Yield coefficient of Nsp | 0.11 | g COD.(g N)^-1^ | [[1](#_ENREF_1), [4](#_ENREF_4)]^(1)^ |
| **Kinetic parameters (pH 7, 28°C)** | | | | |
|  | Nitrite affinity constant of *Nitrobacter* | 1.5 | g N.m^-3^ | [[5](#_ENREF_5)] |
|  | Nitrite affinity constant of *Nitrospira* | 0.12 | g N.m^-3^ | [[1](#_ENREF_1)] |
|  | Oxygen affinity constant of *Nitrobacter* | 0.5 | g O_2_.m^-3^ | [[1](#_ENREF_1)] |
|  | Oxygen affinity constant of *Nitrospira* | 0.9 | g O_2_.m^-3^ | [[1](#_ENREF_1)] |
|  | Maximum growth rate *Nitrobacter* | 1 | d^-1^ | [[6](#_ENREF_6)] |
|  | Maximum growth rate *Nitrospira* | 0.67 | d^-1^ | [[7](#_ENREF_7)] |

1. After unit conversion, using a typical biomass composition of CH_1.8_O_0.5_N_0.2_, corresponding to 1.3659 g COD.g^−1^.

**References**

1. Blackburne R, Vadivelu VM, Yuan ZG, Keller J. Kinetic characterisation of an enriched Nitrospira culture with comparison to Nitrobacter. Water Res. 2007;41(14):3033-42. doi: DOI 10.1016/j.watres.2007.01.043. PubMed PMID: WOS:000248157600001.

2. Blackburne R, Vadivelu VM, Yuan Z, Keller J. Kinetic characterisation of an enriched Nitrospira culture with comparison to Nitrobacter. Water Research. 2007;41(14):3033-42. doi: <http://dx.doi.org/10.1016/j.watres.2007.01.043>.

3. Wiesmann U. Biological nitrogen removal from wastewater. Fiechter, editor Advances in biochemical engineering/biotechnology Springer-Verlag Berlin Heidelberg, Berlin. 1994;1:Pages 113-54.

4. Wiesmann U. Biological nitrogen removal from wastewater. 1994. In: Advances in biochemical engineering/biotechnology [Internet]. Berlin: Springer-Verlag Berlin HeidelbergAdvances in Biochemical Engineering/Biotechnology; [113-54]. Available from: <http://dx.doi.org/10.1007/BFb0008736>.

5. Vadivelu VM, Yuan Z, Fux C, Keller J. Stoichiometric and kinetic characterisation of Nitrobacter in mixed culture by decoupling the growth and energy generation processes. Biotechnology and Bioengineering. 2006;94(6):1176-88. doi: 10.1002/bit.20956.

6. Kindaichi T, Kawano Y, Ito T, Satoh H, Okabe S. Population dynamics and in situ kinetics of nitrifying bacteria in autotrophic nitrifying biofilms as determined by real-time quantitative PCR. Biotechnology and Bioengieering. 2006;94(6):1111–21.

7. Ehrich S, Behrens D, Lebedeva E, Ludwig W, Bock E. A new obligately chemolithoautotrophic, nitrite-oxidizing bacterium,Nitrospira moscoviensis sp. nov. and its phylogenetic relationship. Archives of Microbiology. 1995;164(1):16-23. doi: 10.1007/bf02568729.
