# Supplementary material for: Effect of the dilution rate on microbial competition: r-strategist can win over k-strategist at low substrate concentration
Source: PLoS One. 2017 Mar 23;12(3):e0172785. doi: 10.1371/journal.pone.0172785 (PMC5363889; doi:10.1371/journal.pone.0172785)
Supplement: S5 Table — (DOCX) [file pone.0172785.s005.docx]

**S5** **Table.** Determination of the minimal necessary hydraulic retention time (HRT) to avoid washout in two separate batch reactors containing *Nitrobacter* and *Nitrospira*

| HRT  (days) | Dilution rate  (d^-1^) | Washout *Nitrobacter vulgaris* | Washout *Nitrospira defluvii* |
| --- | --- | --- | --- |
| 2.3 | 0.43 | No | No |
| 2.1 | 0.48 | No | No |
| 1.9 | 0.53 | No | No |
| 1.7 | 0.59 | No | No |
| 1.5 | 0.67 | No | Yes |
